# Development and Initial Validation of the Japanese Version of the Movie for the Assessment of Social Cognition in Adults With Autism Spectrum Disorder

**DOI:** 10.1002/cns.71131

**Published:** 2026-09-01

**Authors:** Muneaki Ogata, Hikaru Hori, Asuka Katsuki, Hitoshi Iida, Leo Gotoh, Haruka Fujino

**Affiliations:** ^1^ Department of Psychiatry, Faculty of Medicine Fukuoka University Fukuoka Japan; ^2^ Nijoufukushikai Social Welfare Corporation Fukuoka Japan; ^3^ Department of Nursing Fukuoka University Hospital Fukuoka Japan

**Keywords:** autism spectrum disorder, MASC, mentalizing, social cognition, theory of mind

## Abstract

**Aims:**

This study aimed to develop a Japanese version of the Movie for the Assessment of Social Cognition (MASC‐J) and to conduct a preliminary evaluation of its reliability and validity in adults with autism spectrum disorder (ASD) compared with healthy controls (HCs).

**Methods:**

Thirty adults with ASD and 30 HCs completed the MASC‐J, Reading the Mind in the Eyes Test (RMET), Autism‐Spectrum Quotient, and Japanese Adult Reading Test. Internal consistency, test–retest reliability, partial convergent validity with the RMET, and discriminative ability using receiver operating characteristic analysis were examined.

**Results:**

MASC‐J scores were significantly lower in the ASD group than in the HC group. The MASC‐J showed acceptable discriminative ability (area under the curve = 0.804), acceptable internal consistency, and excellent test–retest reliability. In the total sample, MASC‐J scores correlated with RMET scores, but this association was not significant within either group. In the ASD group, MASC‐J scores were positively associated with estimated full‐scale IQ. Overmentalizing and absence‐of‐mentalizing errors were more frequent in the ASD group, whereas undermentalizing errors did not differ between groups.

**Conclusion:**

These findings provide preliminary support for the MASC‐J as a measure of social cognition in Japanese‐speaking adults with ASD.

## Graphical abstract

The Japanese version of the Movie for the Assessment of Social Cognition (MASC‐J) showed acceptable internal consistency, excellent test–retest reliability, and acceptable discriminative ability for distinguishing adults with autism spectrum disorder from healthy controls. Adults with autism spectrum disorder showed lower MASC‐J scores and more overmentalizing and absence‐of‐mentalizing errors, providing preliminary support for MASC‐J as a social cognition measure in Japanese‐speaking adults.

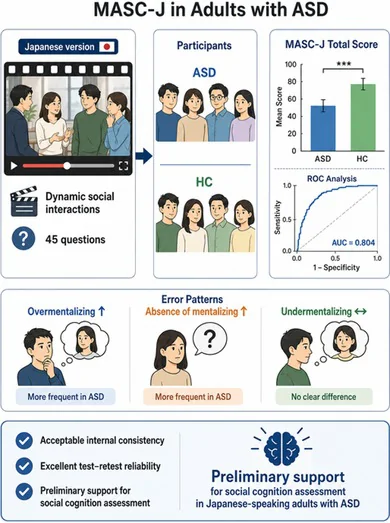

## 1 Introduction

Social cognition refers to the ability to understand others' internal states, such as emotions, intentions, and beliefs, and to adjust one's own behavior accordingly. It is a core cognitive function that underlies effective interpersonal relationships and social adaptation [1]. Social cognition comprises several subdomains, including social perception, emotion recognition, Theory of Mind (ToM), attributional style, and social decision‐making. Impairments in these domains are strongly associated with functional outcomes in psychiatric and neurodevelopmental conditions such as autism spectrum disorder (ASD), schizophrenia, and mood disorders [2, 3, 4]. Among these domains, ToM—the ability to infer others' intentions and beliefs underlying behavior—has long been regarded as a core impairment in ASD [5]. Individuals with ASD often experience difficulties in understanding others' perspectives and implicit social rules, which frequently contribute to interpersonal problems and social isolation [6].

Within the broader construct of social cognition, ToM is often referred to as mentalizing and denotes the capacity to infer others' beliefs, intentions, emotions, and psychological states. Although ToM and mentalizing are not always considered fully synonymous in the literature, they overlap substantially in their shared focus on understanding and inferring others' mental states. In the present study, we therefore use these terms as largely interchangeable when describing the ability assessed by the MASC‐J [7, 8].

ToM has traditionally been assessed using tasks such as classical false‐belief paradigms, the Reading the Mind in the Eyes Test (RMET), and the Hinting Task [9]. These measures have been translated into multiple languages and are widely used in both clinical and research settings. However, many of these tasks rely on static stimuli or highly constrained contexts, raising concerns that they may not fully capture the temporal and contextual complexity inherent in real‐world social interactions [10, 11]. Recent research has therefore emphasized the need for socially and ecologically valid assessments, including tasks that incorporate dynamic stimuli and social situations that more closely approximate naturalistic settings [10, 11]. In addition, cultural differences in emotional expression and its interpretation have been extensively documented [12], underscoring the importance of developing social‐cognitive assessment tools that account for cultural background.

One such tool designed to address these limitations is the Movie for the Assessment of Social Cognition (MASC). The MASC is a video‐based social cognition measure in which participants watch a short film (approximately 15 min long) featuring multiple characters and answer questions sequentially during viewing [13]. By utilizing dynamic stimuli and depicting social situations that resemble everyday interactions, the MASC is considered a ToM task with high ecological validity. The reliability and validity of the MASC have been examined in its original version and in Spanish and Taiwanese adaptations [13, 14, 15]. Studies using the MASC have also been conducted in adolescents with ASD, individuals with borderline personality disorder, and schizophrenia [16, 17, 18]. These findings suggest that the MASC may be a useful tool for assessing social cognition across different clinical populations.

Japanese versions of the RMET and Hinting Task are available; however, a Japanese version of the MASC has yet to be developed. The development of a ToM task that reflects naturalistic social situations is therefore clinically and scientifically important. Accordingly, the present study aimed to develop a Japanese version of the MASC (MASC‐J) and to conduct an initial evaluation of its psychometric properties in adults with ASD and healthy controls (HCs). Specifically, we examined internal consistency, test–retest reliability, partial convergent validity with the RMET, discriminative ability using receiver operating curve (ROC) analysis to distinguish the ASD and HC groups, between‐group differences in mentalizing error patterns, and exploratory associations with sociodemographic factors such as sex and age.

## 2 Methods

### 2.1 Participants

A total of 60 adults participated in the study, including 30 individuals with ASD and 30 HCs. Participants in the ASD group had been diagnosed with ASD by board‐certified psychiatrists in accordance with the Diagnostic and Statistical Manual of Mental Disorders, Fifth Edition [19]. None had comorbid intellectual disability. The HC group was recruited from adult volunteers with no history of psychiatric or neurological disorders. All participants received written and verbal explanations of the study objectives and procedures and provided written informed consent before participation.

The target sample size was determined based on feasibility and with reference to the sample sizes of previous initial validation studies of the original and translated MASC versions [13, 14, 15]. The study was intended as an initial, exploratory evaluation rather than a comprehensive psychometric validation. Additional clinical information, including psychiatric comorbidities and psychotropic medication use, was extracted from available medical records for participants with ASD. Psychiatric comorbidities were categorized as attention‐deficit/hyperactivity disorder, depressive disorders, anxiety disorders, other psychiatric comorbidities, or no comorbidity. Psychotropic medications were categorized as antidepressants, antipsychotics, ADHD medications, anxiolytics/hypnotics, mood stabilizers, or antiepileptic medications. Details are presented in Table 1. These variables were summarized descriptively and were not included as covariates in the primary analyses because of the limited sample size.

**TABLE 1:** Demographic characteristics of the ASD and healthy control groups, and clinical and medication characteristics of the ASD group.

Variable	ASD (*n* = 30)	HC (*n* = 30)	Test statistic	*p*	Effect size/estimate
Age, years	31.50 ± 11.51	33.53 ± 6.71	Z = −1.55	0.122	*r* = 0.20; HL = −4.00, 95% CI = −8.00 to 1.00
Sex, female/male	12/18	14/16	Fisher's exact test	0.795	OR = 1.31, 95% CI = 0.47 to 3.65
JART‐estimated FIQ	109.77 ± 8.18	106.93 ± 7.66	Z = 1.60	0.109	*r* = 0.21; HL = 3.00, 95% CI = −1.00 to 8.00
AQ total score	30.57 ± 7.60	16.17 ± 6.32	Z = 5.70	< 0.001	*r* = 0.74; HL = 15.00, 95% CI = 11.00 to 19.00
Clinical characteristic					
Psychiatric comorbidities					
ADHD	10 (33.3%)				
Depressive disorder	5 (16.7%)				
Other psychiatric comorbidity	1 (3.3%)				
No psychiatric comorbidity	14 (46.7%)				
Psychotropic medication use					
Any psychotropic medication	18 (60.0%)				
Antidepressants	6 (20.0%)				
Antipsychotics	8 (26.7%)				
ADHD medications	7 (23.3%)				
Anxiolytics/hypnotics	10 (33.3%)				
Mood stabilizers/antiepileptic drugs	3 (10.0%)				
No psychotropic medication	12 (40.0%)				

*Note:* Values are presented as mean ± SD unless otherwise indicated. Between‐group comparisons for continuous variables were conducted using the Wilcoxon rank‐sum test. Sex distribution was compared using Fisher's exact test. Effect size r was calculated as |Z|/√N. HL indicates the Hodges–Lehmann estimate of the between‐group difference, calculated as ASD minus HC. For sex distribution, the odds ratio represents the odds of male sex in the ASD group relative to the HC group. Psychiatric comorbidities and psychotropic medication use were assessed only in the ASD group and are presented as *n* (%). Except for the “no psychiatric comorbidity” and “no psychotropic medication” categories, psychiatric comorbidity categories and medication classes were not mutually exclusive because some participants had more than one comorbid condition and/or received medications from multiple classes.

Abbreviations: ADHD, attention‐deficit/hyperactivity disorder; AQ, Autism‐Spectrum Quotient; ASD, autism spectrum disorder; CI, confidence interval; FIQ, full‐scale intelligence quotient; HC, healthy controls; JART, Japanese Adult Reading Test; OR, odds ratio.

### 2.2 Measures

#### 2.2.1 MASC‐J

Permission to develop the Japanese version of the MASC was obtained from the original author. The dialogue, questions, and response options were independently translated into Japanese by four native Japanese‐speaking board‐certified psychiatrists (MO, HH, AK, and HI). The four translations were compared and integrated into a preliminary Japanese version through repeated consensus meetings. Particular attention was paid to preserving the semantic content of the original version, interpersonal relationships among the characters, emotional nuances, and the social‐cognitive intent of each scene and item. The integrated Japanese version was then back‐translated into English by two native English speakers who were unfamiliar with the original MASC. Discrepancies between the original and back‐translated versions were reviewed and resolved through discussion. The revised back‐translation was submitted to and reviewed by the original author. The final version was dubbed into Japanese by four voice actors corresponding to the number of characters.

For cultural adaptation, the setting, narrative structure, interpersonal relationships, flow of dialogue, and questions of the original MASC were retained; no changes were made to its core content or social situations. Only expressions that sounded unnatural in Japanese or were culturally unfamiliar, such as colloquial jokes and idiomatic expressions, were modified. The preliminary MASC‐J was pilot‐tested in 10 healthy adults to assess comprehensibility and cultural appropriateness. Participants provided verbal feedback on the clarity of the Japanese dialogue, meaning of the questions, and response options. Based on this feedback, only typographical corrections and minor wording adjustments were made. Although a formal quantitative assessment of semantic equivalence was not conducted, semantic equivalence was established through independent forward translations, repeated consensus meetings, back‐translation by translators unfamiliar with the original version, comparison with the original, and review by the original author [20].

The MASC consists of a short film (approximately 15 min long) depicting social interactions among several young adults. During the film, participants respond sequentially to 45 multiple‐choice questions. Each question provides response options related to the characters' emotions, intentions, or beliefs, with one correct answer scored as one point. The total score from the first administration was defined as MASC1. Key features of the MASC include: (1) high ecological validity through dynamic video stimuli; (2) a multi‐item structure that permits a fine‐grained assessment of ToM across successive social situations; and (3) the classification of incorrect responses into distinct mentalizing error types [13].

Consistent with the original version, incorrect responses were categorized as follows: (1) overmentalizing, defined as attributing excessively complex mental states; (2) undermentalizing, defined as insufficient attribution of mental states; and (3) absence of mentalizing, defined as minimal consideration of mental states. The frequency of each error type was calculated and used for between‐group comparisons.

#### 2.2.2 RMET

The RMET presents static photographs showing only the eye region of faces and requires participants to select the most appropriate mental state from four descriptors [9]. We used a Japanese translation of the RMET that is available in Japan, and the number of correct responses was recorded as the total score.

#### 2.2.3 Autism‐Spectrum Quotient (AQ)

The AQ is a 50‐item self‐report questionnaire designed to assess autistic traits across five subscales: social skills, attention switching, attention to detail, communication, and imagination [21].

#### 2.2.4 Japanese Adult Reading Test (JART)

The JART is a word‐reading task involving irregular Japanese kanji compounds. It provides an estimate of full‐scale IQ (FIQ) and is widely used as a proxy measure of verbal intelligence in Japanese populations [22].

### 2.3 Procedure

All assessments were conducted individually in a quiet, private room. The administration order was JART, MASC, RMET, and AQ. In the ASD group, the MASC was readministered approximately 4 weeks later to assess test–retest reliability (MASC2). All tasks were administered and scored by trained researchers (MO, HH, and HF) familiar with standardized MASC procedures.

### 2.4 Statistical Analysis

Statistical analyses were performed using JMP software (Student's Edition, version 18.2.2). Descriptive statistics were examined for all variables, and normality was assessed using the Shapiro–Wilk test. Sex distribution was compared using Fisher's exact test. Continuous variables were compared between the ASD and HC groups using the Wilcoxon rank‐sum test. The effect size r for between‐group comparisons was calculated as |Z|/√N, and Hodges–Lehmann estimates with 95% confidence intervals (CIs) were calculated for continuous variables. Internal consistency of the MASC‐J was assessed using Cronbach's alpha.

As a preliminary item‐level descriptive analysis, correct response rates for each MASC‐J item were calculated in the total sample and in each group. Items with correct response rates greater than 90% in the total sample were considered to have potential ceiling effects, whereas items with rates below 10% were considered to have potential floor effects. Considering the limited sample size, these analyses were exploratory. Test–retest reliability in the ASD group was evaluated using the Wilcoxon signed‐rank test to compare MASC1 and MASC2 scores and by calculating intraclass correlation coefficients (ICCs).

To examine partial convergent validity, Pearson correlation coefficients with 95% CIs were calculated between MASC1 and RMET or JART scores in the total sample and separately within each group. Discriminative ability was evaluated using ROC analysis, with diagnostic group (ASD vs. HC) as the outcome and MASC1 as the predictor. AUCs with 95% CIs, optimal cutoff values, sensitivity, specificity, and Youden indices were calculated. The optimal cutoff was determined using the Youden index. ROC analysis was also performed for the RMET.

Multiple linear regression analyses were conducted separately in the ASD and HC groups. MASC1 was entered as the dependent variable, and age, sex, JART, RMET, and AQ scores were entered as independent variables. Unstandardized coefficients, standard errors, standardized coefficients, 95% CIs, *p* values, and variance inflation factors were calculated. Considering the limited group‐specific sample sizes and number of predictors, these regression analyses were considered exploratory and interpreted cautiously. All tests were two‐sided, with *p* < 0.05 being considered statistically significant.

## 3 Results

Descriptive statistics for the ASD (*n* = 30) and HC (*n* = 30) groups are presented in Table 1. No significant between‐group differences were observed in age, estimated FIQ, or sex distribution. In contrast, AQ scores were significantly higher in the ASD group (Z = 5.70, *p* < 0.001, *r* = 0.74), indicating higher levels of autistic traits. Psychiatric comorbidities and psychotropic medication use in the ASD group are also summarized in Table 1.

As an exploratory descriptive analysis within the ASD group, MASC‐J total scores were compared according to psychotropic medication use, psychiatric comorbidity status, and antipsychotic use. MASC‐J total scores did not differ significantly between participants with and without psychotropic medication use (26.94 ± 4.80 vs. 27.17 ± 7.71, *p* = 0.432), psychiatric comorbidity (27.69 ± 5.11 vs. 26.29 ± 7.01, *p* = 0.754), or antipsychotic use (27.38 ± 4.24 vs. 26.91 ± 6.61, *p* = 0.851).

Descriptive statistics for MASC‐J scores, RMET scores, and mentalizing error types are shown in Table 2. MASC‐J scores were significantly higher in the HC group than in the ASD group (Z = −4.05, *p* < 0.001, *r* = 0.52). RMET scores were also significantly higher in the HC group (Z = −2.34, *p* = 0.019, *r* = 0.30). Individuals with ASD made significantly more overmentalizing errors (Z = 2.47, *p* = 0.013, *r* = 0.32) and absence‐of‐mentalizing errors (Z = 4.23, *p* < 0.001, *r* = 0.55), whereas undermentalizing errors did not differ significantly between groups (Z = 1.33, *p* = 0.184, *r* = 0.17).

**TABLE 2:** Group comparisons of MASC‐J, RMET, and mentalizing error scores.

Variable	ASD (*n* = 30)	HC (*n* = 30)	Z	*p*	Effect size r	HL difference (95% CI)
MASC‐J total score	27.03 ± 6.00	32.37 ± 3.22	−4.05	< 0.001	0.52	−4.00 (−7.00 to −2.00)
RMET total score	20.20 ± 2.20	21.87 ± 2.81	−2.34	0.019	0.30	−2.00 (−3.00 to 0.00)
Overmentalizing errors	6.37 ± 3.13	4.57 ± 2.19	2.47	0.013	0.32	2.00 (0.00 to 3.00)
Undermentalizing errors	5.90 ± 2.63	5.00 ± 1.89	1.33	0.184	0.17	1.00 (0.00 to 2.00)
Absence‐of‐mentalizing errors	5.37 ± 2.40	3.07 ± 1.51	4.23	< 0.001	0.55	2.00 (1.00 to 3.00)

*Note:* Values are presented as mean ± SD. Between‐group comparisons were conducted using the Wilcoxon rank‐sum test. Effect size r was calculated as |Z|/√N. HL difference represents the Hodges–Lehmann estimate of the between‐group difference, calculated as ASD minus HC.

Abbreviations: ASD, autism spectrum disorder; CI, confidence interval; HC, healthy controls; MASC‐J, Movie for the Assessment of Social Cognition–Japanese version; RMET, Reading the Mind in the Eyes Test.

Internal consistency of MASC1 was acceptable (Cronbach's alpha = 0.769). Test–retest reliability was assessed in 29 participants with ASD who completed both administrations. MASC1 and MASC2 scores were strongly positively correlated (*r* = 0.95, *p* < 0.001), and the ICC indicated excellent agreement (ICC = 0.95). One participant was excluded from the test–retest analysis because MASC2 data were missing. Preliminary item‐level descriptive analyses, including correct response rates and potential ceiling and floor effects, are presented in Table S1. Several items showed relatively low correct‐response rates, but these items were retained because the overall internal consistency of the MASC‐J was acceptable and the total score showed significant group differences between the ASD and HC groups.

As shown in Table 3, MASC‐J total scores were significantly and positively correlated with RMET performance in the total sample (*r* = 0.26, 95% CI 0.006 to 0.482, *p* = 0.045). Conversely, no significant association was observed with JART‐derived FIQ (*r* = 0.19, 95% CI −0.064 to 0.426, *p* = 0.139). In the HC group, MASC‐J scores were not significantly correlated with RMET (*r* = 0.06, 95% CI −0.311 to 0.407, *p* = 0.773) or JART (*r* = 0.04, 95% CI −0.325 to 0.395, *p* = 0.833). In the ASD group, MASC‐J scores were not significantly correlated with RMET (*r* = 0.19, 95% CI −0.180 to 0.517, *p* = 0.308) but were positively correlated with JART (*r* = 0.49, 95% CI 0.157 to 0.722, *p* = 0.006).

**TABLE 3:** Correlations between MASC‐J scores and comparator measures.

Sample	Comparator measure	r	95% CI for r	*p*
Total sample	RMET	0.26	0.006 to 0.482	0.045
ASD group	RMET	0.19	−0.180 to 0.517	0.308
HC group	RMET	0.06	−0.311 to 0.407	0.773
Total sample	JART‐estimated FIQ	0.19	−0.064 to 0.426	0.139
ASD group	JART‐estimated FIQ	0.49	0.157 to 0.722	0.006
HC group	JART‐estimated FIQ	0.04	−0.325 to 0.395	0.833

*Note:* Pearson's correlation coefficients are shown.

Abbreviations: ASD, autism spectrum disorder; CI, confidence interval; FIQ, full‐scale intelligence quotient; HC, healthy controls; JART, Japanese Adult Reading Test; MASC‐J, Movie for the Assessment of Social Cognition–Japanese version; RMET, Reading the Mind in the Eyes Test.

**FIGURE 1:**
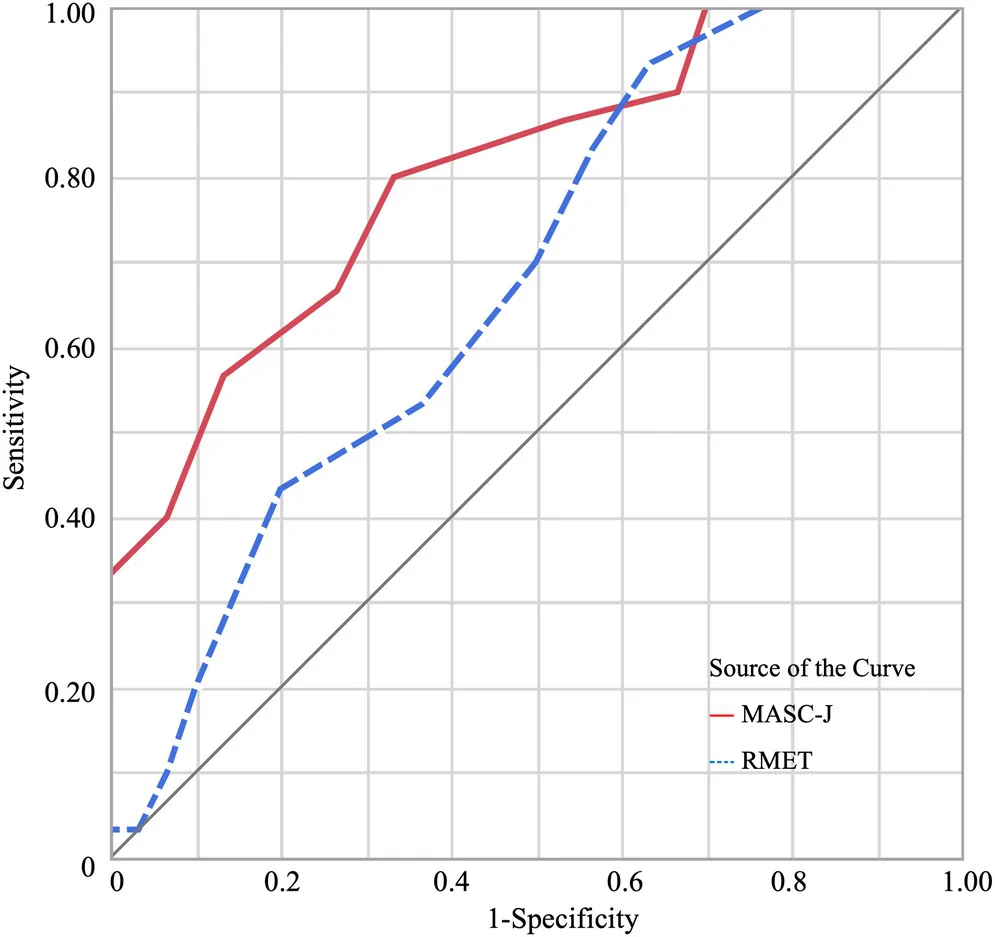
Receiver operating characteristic curves for the MASC‐J and RMET. The diagonal line represents chance performance. The area under the curve was 0.804 for the MASC‐J and 0.675 for the RMET. MASC‐J, Movie for the Assessment of Social Cognition–Japanese version; RMET, Reading the Mind in the Eyes Test.

ROC analysis was performed to evaluate the ability of MASC‐J total scores to distinguish participants with ASD from HCs (Figure 1; Table 4). When ASD was designated as the positive condition, the AUC was 0.804 (95% CI 0.674–0.891). The optimal cutoff value was 31, with a sensitivity of 0.80 and specificity of 0.67. Diagnostic performance at each candidate MASC‐J cutoff is presented in Table S2. In exploratory multiple regression analyses (Table 5), none of the variables significantly predicted MASC1 performance in the HC group. In contrast, JART was the only variable significantly associated with MASC1 scores in the ASD group.

**TABLE 4:** ROC analysis for distinguishing ASD from HC participants.

Measure	AUC	95% CI for AUC	Optimal cut‐off	Sensitivity	Specificity	Youden index
MASC‐J	0.804	0.674 to 0.891	31	0.80	0.67	0.47
RMET	0.675	0.527 to 0.795	23	0.93	0.37	0.30

*Note:* ASD was treated as the positive class. For each measure, participants with scores at or below the optimal cut‐off were classified as ASD, whereas those with scores above the cut‐off were classified as HC. The Youden index was calculated as sensitivity + specificity −1.

Abbreviations: ASD, autism spectrum disorder; AUC, area under the receiver operating characteristic curve; CI, confidence interval; HC, healthy controls; MASC‐J, Movie for the Assessment of Social Cognition–Japanese version; RMET, Reading the Mind in the Eyes Test.

**TABLE 5:** Exploratory multiple regression analyses predicting MASC‐J total score.

Group	Predictor	B	SE	β	95% CI for B	*p*	VIF
HC	Age	−0.027	0.099	−0.057	−0.232 to 0.178	0.785	1.05
	Female sex	0.079	0.689	0.025	−1.342 to 1.500	0.909	1.15
	JART‐estimated FIQ	0.021	0.093	0.051	−0.170 to 0.213	0.820	1.19
	RMET	0.003	0.254	0.003	−0.521 to 0.527	0.991	1.20
	AQ	−0.057	0.113	−0.113	−0.291 to 0.176	0.616	1.20
ASD	Age	−0.048	0.101	−0.091	−0.255 to 0.160	0.640	1.20
	Female sex	0.553	1.063	0.092	−1.641 to 2.747	0.608	1.01
	JART‐estimated FIQ	0.384	0.150	0.523	0.074 to 0.693	0.017	1.35
	RMET	0.175	0.520	0.064	−0.898 to 1.249	0.739	1.18
	AQ	0.042	0.150	0.053	−0.267 to 0.350	0.783	1.16

*Note:* MASC‐J total score was entered as the dependent variable. Age, sex, JART‐estimated FIQ, RMET score, and AQ score were entered simultaneously as independent variables. Sex was coded as 0 = male and 1 = female. These regression analyses were exploratory because of the modest subgroup sample sizes. HC model: R^2^ = 0.017, adjusted R^2^ = −0.188, F(5, 24) = 0.084, *p* = 0.994. ASD model: R^2^ = 0.258, adjusted R^2^ = 0.103, F(5, 24) = 1.667, *p* = 0.181.

Abbreviations: AQ, Autism‐Spectrum Quotient; ASD, autism spectrum disorder; B, unstandardized regression coefficient; CI, confidence interval; FIQ, full‐scale intelligence quotient; HC, healthy controls; JART, Japanese Adult Reading Test; MASC‐J, Movie for the Assessment of Social Cognition–Japanese version; RMET, Reading the Mind in the Eyes Test; SE, standard error; VIF, variance inflation factor; β, standardized regression coefficient.

## 4 Discussion

The present study developed the MASC‐J and conducted an initial evaluation of its reliability, validity, and mentalizing error patterns in adults with ASD and HCs. However, because multiple statistical analyses were conducted without formal correction for multiple comparisons, significant findings, particularly those from secondary and subgroup analyses, should be interpreted cautiously.

The MASC‐J demonstrated acceptable internal consistency and excellent test–retest reliability, suggesting that it may provide a relatively stable assessment of social cognition in adults with ASD without intellectual disability. These findings are consistent with those reported for the original and other language versions [13, 14, 15]. MASC‐J scores were significantly lower in the ASD group than the HC group, and ROC analysis yielded an AUC of 0.804, indicating acceptable discriminative ability. These findings are broadly consistent with previous reports on the original, Spanish, and Taiwanese versions of the MASC [13, 14, 15]. However, the AUC observed in the present study was somewhat lower than those reported previously. One possible explanation is the clinical and cognitive heterogeneity in the ASD group. ASD is characterized by substantial interindividual variability in cognitive and social functioning. This variability may have increased as diagnostic criteria broadened and more diverse clinical profiles were included in research [23]. Greater variability may increase the overlap in score distributions between the ASD and HC groups, thereby affecting discriminative ability. However, the effects of clinical and cognitive heterogeneity on MASC‐J scores and AUC were not directly examined in this study.

The significant association between MASC‐J and RMET scores in the total sample provides partial support for convergent validity. However, although the RMET assesses a related aspect of social cognition, the two tasks do not measure the same ability. The RMET primarily assesses the inference of mental states from static images of the eye region [9]. In contrast, the MASC‐J requires integration of information about dialogue, emotion, interpersonal relationships, and the unfolding context of social interaction. Accordingly, the two tasks likely share certain aspects of social cognition while involving different cognitive processes [10, 11].

The error‐pattern analysis showed that the ASD group made more overmentalizing and absence‐of‐mentalizing errors than the HC group, whereas undermentalizing errors did not differ significantly between the groups. These findings suggest that social‐cognitive difficulties in adults with ASD may not be captured by a single mentalizing error type and that response tendencies may vary according to social context, task demands, and the information available in a given scene. MASC‐J scores were also positively associated with JART‐estimated FIQ in the ASD group, suggesting that verbal reasoning or general cognitive abilities may contribute to processing the complex social situations assessed by the MASC‐J. These findings may be consistent with the concept of cognitive compensation in ASD [24]. However, this interpretation requires caution. The present study examined error frequencies from a single MASC‐J administration at one time point and therefore cannot establish disorder‐specific mentalizing styles, context‐dependent response variability, or psychological and cognitive mechanisms such as cognitive compensation. Longitudinal studies incorporating multiple social‐cognition tasks, neurocognitive measures, and real‐world social functioning are needed to investigate these possibilities.

From a cultural perspective, the MASC‐J was developed to preserve the content of the original version while rendering the dialogue natural and understandable for Japanese‐speaking adults. Some expressions were adapted to improve clarity in Japanese without altering the settings, interpersonal relationships, or information necessary to infer the characters' thoughts and feelings. The present findings provide preliminary support for the feasibility of administering the MASC‐J to Japanese‐speaking adults. However, cultural appropriateness, semantic equivalence, and psychometric properties require further evaluation.

Clinically, the MASC‐J may be useful as a research and assessment tool for examining social cognition in Japanese‐speaking adults with ASD. However, the present findings should not be interpreted as establishing its diagnostic or screening utility or its applicability to other psychiatric disorders. Overall, the findings provide preliminary support for the reliability and validity of the MASC‐J as a measure of social cognition in Japanese‐speaking adults with ASD. Future studies should examine its stability, dimensional structure, relationships with other social‐cognition tasks and real‐world social functioning, and potential utility in populations beyond ASD. The MASC‐J may eventually contribute to individualized intervention planning for social cognition, including interventions such as Social Cognition and Interaction Training and Mentalization‐Based Treatment [25, 26].

## 5 Limitations

Several limitations should be acknowledged. First, although internal consistency and test–retest reliability were evaluated, the sample size was insufficient for a comprehensive psychometric evaluation of the 45‐item MASC‐J. At the item level, correct response rates were described and potential ceiling and floor effects were examined preliminarily. However, item‐total correlations and response distributions across individual answer options were not analyzed. In addition, factor structure and dimensionality were not evaluated using exploratory or confirmatory factor analysis; item characteristics were not examined using Rasch modeling or item response theory; and measurement invariance between the ASD and HC groups was not assessed. Therefore, Cronbach's alpha should be interpreted only as an index of internal consistency and not as evidence that the MASC‐J is unidimensional or reflects a single common ability. In addition, the excellent test–retest reliability observed over the 4‐week interval should be interpreted cautiously. Because the MASC‐J uses fixed video content and questions, participants may have remembered the story, questions, or their previous responses at the second administration. Therefore, the high ICC may partly reflect memory effects. Future studies should examine test–retest reliability using longer retest intervals or approaches less susceptible to memory effects.

Second, multiple statistical analyses were performed without formal adjustment for multiple comparisons. Consequently, some significant findings, including those from subgroup and secondary analyses, may represent chance findings and should be interpreted cautiously as exploratory findings.

Third, the RMET was the only comparison measure used to evaluate convergent validity because standardized Japanese measures of adult social cognition are limited. However, the RMET assesses a relatively restricted and static aspect of social cognition and differs from the MASC‐J in stimulus format and task demands. Thus, the association between MASC‐J and RMET scores provides only partial support for convergent validity. In addition, the order of task administration was fixed rather than counterbalanced. Because all participants completed the MASC‐J before the RMET, fatigue after the 45‐item MASC‐J may have affected subsequent RMET performance and weakened the observed association between the two measures. Future studies should counterbalance the order of task administration to reduce potential fatigue effects related to fixed task order.

Fourth, intellectual functioning was estimated using the JART, which is based on acquired verbal abilities and may not accurately reflect current overall cognitive functioning.

Fifth, although the MASC‐J was developed through translation, back‐translation, consensus procedures, dubbing, pilot testing, and review by the original author, formal quantitative evaluation of semantic equivalence between the original and Japanese versions was not performed.

Finally, the ASD group consisted primarily of adults without intellectual disability and included participants with psychiatric comorbidities and psychotropic medication use. Although exploratory descriptive analyses did not show significant differences in MASC‐J scores according to psychotropic medication use, psychiatric comorbidity status, or antipsychotic use within the ASD group, these findings should be interpreted cautiously because of the small subgroup sizes and limited statistical power. Future studies with larger samples should more systematically evaluate the effects of medication and comorbidity on MASC‐J performance. In addition, educational attainment, socioeconomic background, ASD severity or support level, and occupational functioning were not systematically collected. Caution is therefore warranted when generalizing the findings to individuals with ASD and intellectual disability, children, adolescents, or older adults.

## 6 Conclusion

The present study developed a Japanese version of the MASC and conducted an initial evaluation of its psychometric properties in adults with ASD and HCs. The MASC‐J showed acceptable internal consistency, excellent test–retest reliability, and an association with the RMET that provides partial support for convergent validity. It also demonstrated acceptable discriminative ability for distinguishing adults with ASD from HCs in this sample. Overall, these findings provide preliminary support for the MASC‐J as a measure of social cognition in Japanese‐speaking adults with ASD. Larger and more diverse samples are needed to confirm its psychometric properties, dimensional structure, and clinical utility.

## Author Contributions

Muneaki Ogata and Hikaru Hori contributed to the conception and design of the study. Muneaki Ogata, Hikaru Hori, Asuka Katsuki, and Hitoshi Iida conducted the translation process and data collection. Muneaki Ogata, Hikaru Hori, Asuka Katsuki, Hitoshi Iida, and Haruka Fujino contributed to participant recruitment, and Muneaki Ogata, Hikaru Hori, and Haruka Fujino conducted the assessments. Muneaki Ogata and Leo Gotoh performed the statistical analyses. Muneaki Ogata drafted the manuscript. All authors critically revised the manuscript for important intellectual content, approved the final version, and agree to be accountable for all aspects of the work.

## Funding

This study was supported by a grant from the Clinical Research Promotion Foundation (2024) awarded to Haruka Fujino and the Japan Society for the Promotion of Science (JSPS KAKENHI Grant Number 26K20553) awarded to Hitoshi Iida.

## Disclosure

The authors have nothing to report.

## Ethics Statement

The study protocol was approved by the Ethics Committee for Research Involving Human Subjects at Fukuoka University (approval number: U24‐06‐008) and was conducted in accordance with the Declaration of Helsinki.

## Conflicts of Interest

The authors declare no conflicts of interest.

## Supporting information


**Table S1:** Item‐level correct response rates for the MASC‐J.
**Table S2:** Classification performance of MASC‐J total scores across candidate cut‐off values for distinguishing adults with ASD from healthy controls.

## Data Availability

The data supporting the findings of this study are not publicly available because of ethical and privacy restrictions. The informed consent obtained from participants did not include permission for public sharing of individual‐level data.
