# Supplementary material for: Development and Initial Validation of the Japanese Version of the Movie for the Assessment of Social Cognition in Adults With Autism Spectrum Disorder
Source: CNS Neurosci Ther. 2026 Sep 1;32(9):e71131. doi: 10.1002/cns.71131 (PMC13532569; doi:10.1002/cns.71131)
Supplement: Supplementary file 1 [file CNS-32-e71131-s001.docx]

Supplementary Material

Supplementary Table 1. Item-level correct response rates for the MASC-J

| **Item** | **Total sample (%)** | **ASD group (%)** | **HC group (%)** |
| --- | --- | --- | --- |
| 1 | 66.7 | 60.0 | 73.3 |
| 2 | 91.7 | 86.7 | 96.7 |
| 3 | 46.7 | 40.0 | 53.3 |
| 4 | 26.7 | 13.3 | 40.0 |
| 5 | 60.0 | 46.7 | 73.3 |
| 6 | 71.7 | 70.0 | 73.3 |
| 7 | 76.7 | 70.0 | 83.3 |
| 8 | 70.0 | 63.3 | 76.7 |
| 9 | 48.3 | 36.7 | 60.0 |
| 10 | 85.0 | 83.3 | 86.7 |
| 11 | 93.3 | 90.0 | 96.7 |
| 12 | 30.0 | 13.3 | 46.7 |
| 13 | 55.0 | 53.3 | 56.7 |
| 14 | 85.0 | 76.7 | 93.3 |
| 15 | 65.0 | 53.3 | 76.7 |
| 16 | 78.3 | 63.3 | 93.3 |
| 17 | 68.3 | 53.3 | 83.3 |
| 18 | 85.0 | 73.3 | 96.7 |
| 19 | 68.3 | 66.7 | 70.0 |
| 20 | 80.0 | 80.0 | 80.0 |
| 21 | 55.0 | 53.3 | 56.7 |
| 22 | 81.7 | 83.3 | 80.0 |
| 23 | 58.3 | 50.0 | 66.7 |
| 24 | 71.7 | 60.0 | 83.3 |
| 25 | 88.3 | 80.0 | 96.7 |
| 26 | 61.7 | 56.7 | 66.7 |
| 27 | 88.3 | 80.0 | 96.7 |
| 28 | 83.3 | 86.7 | 80.0 |
| 29 | 65.0 | 63.3 | 66.7 |
| 30 | 80.0 | 70.0 | 90.0 |
| 31 | 38.3 | 33.3 | 43.3 |
| 32 | 61.7 | 70.0 | 53.3 |
| 33 | 91.7 | 83.3 | 100.0 |
| 34 | 15.0 | 13.3 | 16.7 |
| 35 | 51.7 | 43.3 | 60.0 |
| 36 | 83.3 | 80.0 | 86.7 |
| 37 | 50.0 | 50.0 | 50.0 |
| 38 | 33.3 | 16.7 | 50.0 |
| 39 | 35.0 | 40.0 | 30.0 |
| 40 | 66.7 | 56.7 | 76.7 |
| 41 | 86.7 | 83.3 | 90.0 |
| 42 | 23.3 | 20.0 | 26.7 |
| 43 | 86.7 | 86.7 | 86.7 |
| 44 | 93.3 | 93.3 | 93.3 |
| 45 | 73.3 | 56.7 | 90.0 |

Note. Correct response rates were calculated for each MASC-J item in the total sample (N = 60), ASD group (n = 30), and HC group (n = 30). Values are presented as percentages. Items with correct response rates above 90% in the total sample were considered to show possible ceiling effects, whereas items with correct response rates below 10% were considered to show possible floor effects. These item-level analyses were exploratory because of the modest sample size. MASC-J, Movie for the Assessment of Social Cognition–Japanese version; ASD, autism spectrum disorder; HC, healthy controls.

Supplementary Table 2.Classification performance of MASC-J total scores across candidate cut-off values for distinguishing adults with ASD from healthy controls

| Cut-off score | Se | Sp | PPV | NPV |
| --- | --- | --- | --- | --- |
| 20 | 0.10 | 1.00 | 1.00 | 0.53 |
| 21 | 0.13 | 1.00 | 1.00 | 0.53 |
| 23 | 0.20 | 1.00 | 1.00 | 0.56 |
| 24 | 0.23 | 1.00 | 1.00 | 0.57 |
| 25 | 0.30 | 1.00 | 1.00 | 0.59 |
| 26 | 0.33 | 1.00 | 1.00 | 0.60 |
| 27 | 0.40 | 0.93 | 0.86 | 0.61 |
| 28 | 0.57 | 0.87 | 0.81 | 0.67 |
| 29 | 0.67 | 0.73 | 0.71 | 0.69 |
| 30 | 0.73 | 0.70 | 0.71 | 0.72 |
| 31 | 0.80 | 0.67 | 0.71 | 0.77 |
| 32 | 0.87 | 0.47 | 0.62 | 0.78 |
| 33 | 0.90 | 0.33 | 0.57 | 0.77 |
| 34 | 1.00 | 0.30 | 0.59 | 1.00 |
| 35 | 1.00 | 0.20 | 0.56 | 1.00 |
| 36 | 1.00 | 0.10 | 0.53 | 1.00 |
| 37 | 1.00 | 0.03 | 0.51 | 1.00 |

Note. ASD was treated as the positive class. For each candidate cut-off score, participants with MASC-J scores at or below the cut-off were classified as ASD, whereas those with scores above the cut-off were classified as healthy controls. Sensitivity (Se) indicates the proportion of participants with ASD correctly classified as ASD, whereas specificity (Sp) indicates the proportion of healthy controls correctly classified as healthy controls. Positive predictive value (PPV) indicates the probability that a participant classified as ASD truly had ASD, and negative predictive value (NPV) indicates the probability that a participant classified as a healthy control truly belonged to the healthy control group. The cut-off score of 31, which maximized the Youden index, was selected as the optimal cut-off value in the present sample. Because the ASD prevalence was 50% in this case–control sample, PPV and NPV are sample-dependent and should not be generalized directly to clinical settings with different ASD prevalence. MASC-J, Movie for the Assessment of Social Cognition–Japanese version; ASD, autism spectrum disorder.
